# Acute Liver Failure Associated with Levetiracetam and Lacosamide Combination Treatment for Unspecified Epileptic Disorder

**DOI:** 10.1155/2013/634174

**Published:** 2013-08-20

**Authors:** Ylse Gutiérrez-Grobe, Jose Antonio Bahena-Gonzalez, Magali Herrera-Gomar, Pedro Mendoza-Diaz, Sandra García-López, Octavio González-Chon

**Affiliations:** ^1^Internal Medicine Department, Medica Sur Clinic and Foundation, Puente de Piedra No. 150, Colonia Toriello Guerra, Delegación Tlalpan, 14050 Mexico City, Mexico; ^2^Coronary Care Unit, Medica Sur Clinic and Foundation, Puente de Piedra No. 150, Colonia Toriello Guerra, Delegación Tlalpan, 14050 Mexico City, Mexico

## Abstract

*Background and Aim*. Levetiracetam is a second-generation antiepileptic drug. It is approved as an adjunctive treatment of partial onset seizures with or without secondary generalization. It is considered safe with less than 1% of patients with transient elevations of liver enzymes. *Methods*. We report a case of acute liver failure secondary to Levetiracetam in combination with Lacosamide documented with a liver biopsy. *Results*. Liver biopsy demonstrated acute liver injury with a predominant submassive necrosis pattern and features of a drug-induced hepatitis. *Conclusions*. This is the first published case of acute liver failure due to antiepileptic therapy with Levetiracetam in combination with Lacosamide.

## 1. Introduction 

Levetiracetam is an established second-generation antiepileptic drug that is approved as a treatment of partial seizures; other indications include adjunctive treatment of myoclonic seizures associated with juvenile myoclonic epilepsy and primary generalized tonic-clonic seizures associated with generalized epilepsy. In vitro and in vivo studies have shown that levetiracetam does not act as a cytochrome P450 inductor; therefore it does not cause liver function alterations. A previous case report suggested that it can slightly raise gamma glutamyl transpeptidase and transaminases, but the indication of it as first-line treatment for patients with epilepsy and liver damage should not be modified, but liver function tests should be monitorized periodically in patients taking this medication [1]. Levetiracetam lacks cytochrome P450 isoenzyme-inducing potential, and no significant interaction with other drugs, including other antiepileptic drugs, has been described.

On the other side, Lacosamide, previously known as Erlosamide, is an adjunctive treatment for epilepsy as well as monotherapy for diabetic neuropathic pain. It appears to have a dual mode of action: selective enhancement of sodium channel inactivation and modulation of collapsin response mediator protein 2. Several trials have demonstrated a reduction in median seizure frequency compared with placebo [2]. Zaccara et al. found, in their study carried out recently, that Lacosamide treatment is associated with symptoms suggestive of vestibulecerebellar dysfunction that worsens with increasing dose without other severe events [3]. Recent data supports this, suggesting that Lacosamide is a safe treatment in critically ill patients, and there is only one report of elevation of liver function tests in a recent retrospective study [4]. 

Herein we report a case of acute liver failure with secondary multiorganic failure associated with Levetiracetam and Lacosamide treatment in a patient with unknown etiology seizures. 

## 2. Case Presentation 

**Figure 1:**
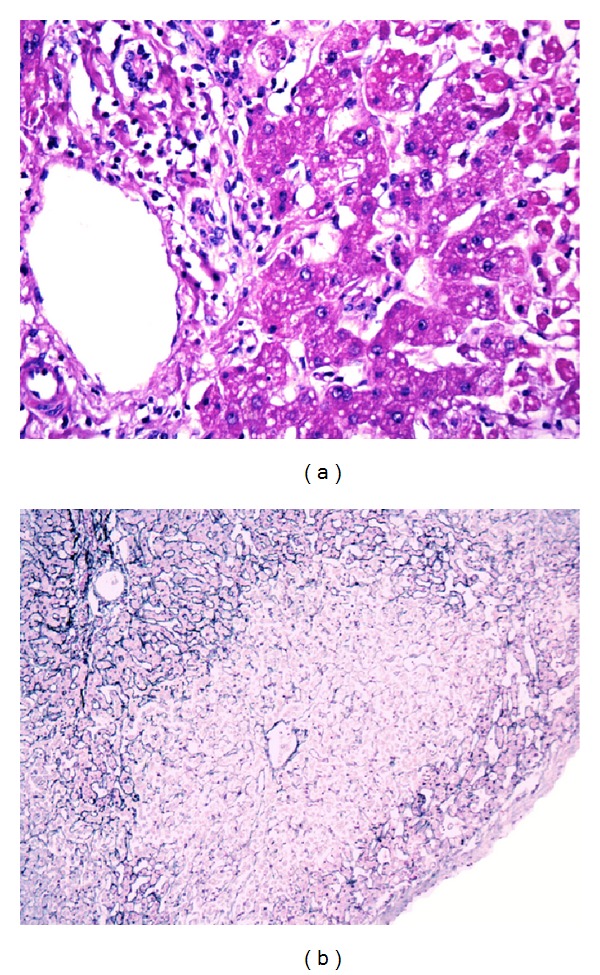
Confluent centrilobular necrosis with reticulin collapse, as well as inflamed portal tracts with and active necroinflammatory tissue. (a) Hematoxylin eosin staining of the transition zone. (b) Gomori silver staining that shows central necrosis in a panoramic view.

A 22-year-old woman was admitted to the Emergency department of Medica Sur Clinic and Foundation because of dizziness, syncope, and convulsive crisis. She has the previous diagnosis of seizures of unknown etiology two years ago, treated recently 2 months before hospitalization by a neurologist with lacosamide at a previous dose of 100 mg twice a day, in combination with levetiracetam 500 mg twice a day. Four days before her admission to the hospital, the antiepileptic drugs doses were reduced at half, this means 50 mg twice a day and 250 mg twice a day, respectively. At that time, general laboratory test was assessed; in these, liver transaminases and serum bilirubin were normal. She also takes Alprazolam 2 mg a day in case of insomnia and no other drugs. The day before her admission she complained of dizziness; she had syncope and then presented with status epilepticus, with ten continuous episodes of generalized tonic-clonic convulsions of seconds to five minutes of duration, followed by disorientation and sleepiness; for this reason she doubled the Levetiracetam dose; in spite of this, she continued with convulsive crisis; subsequently she presented to the emergency room of our hospital. On admission she was sleepy, with a GCS of 8, poor response to external stimulation, pain in upper right quadrant of the abdomen, and tender and enlarged liver without splenomegaly. Laboratory values were as follows: white blood cells 26.8 G/L, platelets 180 G/L, glucose 124 mg/dL, BUN 31.2 mg/dL, creatinine 5.12 mg/dL, albumin 33 g/dL, serum bilirubin 2.14 mg/dL, alanine aminotransferase 4341 IU/L, aspartate aminotransferase 10387 IU/L, alkaline phosphatase 74 IU/L, gamma glutamyl transpeptidase 28 IU/L, creatin phosphokinase 2989 IU/L, mioglobin 14598.7 ng/mL, creatin phosphokinase-MB 43.5 ng/mL, troponin I 3.73 ng/mL, brain natriuretic peptide 3147.6 pg/mL, C-reactive protein 32.4 mg/L, and arterial blood gases with metabolic acidosis: pH 7.16, PCO_2_ 27.4, PO_2_ 107, and lactate 10. EKG with ST elevation in the anteroseptal and inferior walls, transesophageal echocardiography, showed right cavities dilation, severe tricuspid insufficiency, and moderate pulmonary hypertension; for this reason she was admitted to the Coronary Care Unit with vasopressor support with norepinephrine. Pulmonary angiography CT discarded pulmonary thromboembolism. Viral serology for cytomegalovirus, Epstein Barr virus, and hepatitides A, B, C, and E was negative. Immunodeficiency virus serology, blood cultures, and screening for autoantibodies were also negative. The abdominal ultrasonography showed free abdominal fluid without obvious solid organ injury; therefore on day 2 an exploratory laparotomy was carried out, changes in hepatic parenchyma were found, and then a liver biopsy was taken. After the surgery she remained on invasive ventilatory support for 10 days. Histology showed confluent centrilobular necrosis with reticulin collapse, as well as inflamed portal tracts with numerous polynuclear eosinophils, and revealed active necroinflammatory tissue that comprised 85% of liver tissue diagnosing sub massive hepatitis with characteristics of drug-induced liver damage (Figure 1). On July 30, total bilirubin level reached the highest levels, and artificial extracorporeal liver support was carried out for unique occasion. On the 10 day of mechanical ventilation it was suspended with an adequate tolerance. 

**Figure 2:**
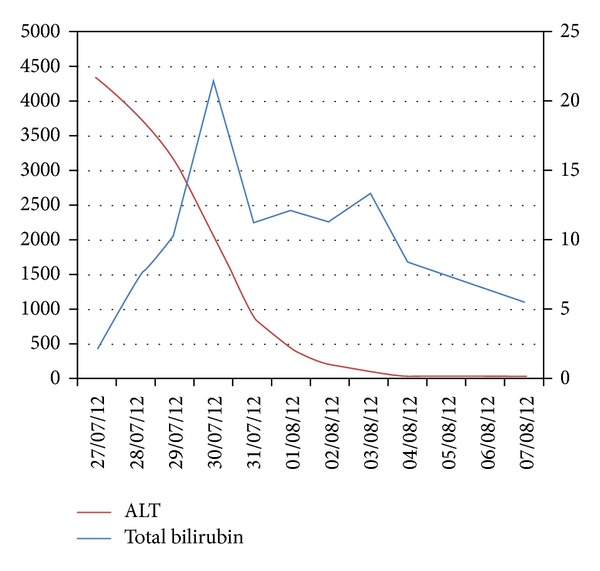
Graphical illustration of ALT (on the left axis) and serum bilirubin (on the right axis) over time.

Serum transaminases were normal on August 7, 12 days after the onset of acute hepatitis.  Figure 2 illustrates the time course of bilirubin and ALT alterations. During her stay in the Coronary Care Unit, the patient gradually improved clinically and biologically over the following days and was discharged from hospital on August 10. 

## 3. Discussion 

That Levetiracetam and Lacosamide combination that appears responsible for the observed liver injury seems quite convincing based on the temporal relationship between administration of the drug and the onset of clinical symptoms, the marked transaminases level elevation in combination with the histologic findings, the absence of alternative diagnosis, and the significant improvement with discontinuation of the drug. To assess the likelihood of a causal association between the medications exposure and the acute hepatitis, we utilized drug-induced liver injury diagnostic scales. Thus, the strength of association between the implicated antiviral and the acute hepatocellular damage was graded as “probable” using the Roussel Uclaf Causality Assessment Method (CIOMS/RUCAM) scale (score of 8) [5] and “possible” using Maria and Victorino scale (score of 12) [6]. The elements missing for a closer association measured by these scales included the absence of a previous report of hepatotoxicity due to Levetiracetam and Lacosamide interaction, although an internet database of adverse events reports one isolated event of this drug interaction associated with acute liver failure. On the other side Tan et al. reported in 2008 Levetiracetam as a possible cause of acute liver failure in a 21-year-old patient that developed fulminant liver failure that subsequently required a liver transplantation. In the case we report that our patient improved significantly with the interruption of the medications and a unique artificial liver support session. It is worth mentioning that creatinine levels in our patients improved only slightly and the patient was dismissed of the hospital with a rigorous follow-up strategy with the nephrologist and the gastroenterologist. The acute kidney failure might have been caused by multiorganic failure, and the patient's current kidney function is normal, with normal values of creatinine. 

Interestingly, liver biopsy is not routinely recommended when drug-induced liver injury is suspected. However, when it is performed, it provides important information on the pattern of liver injury and demonstrates histological features suggestive of drug-induced liver injury. In this patient, the pattern was predominantly cytolytic and highly suggestive of a toxic mechanism.
